# Air pollution-related deaths in Europe – time for action

**DOI:** 10.7189/jogh.09.020308

**Published:** 2019-12

**Authors:** Helotonio Carvalho

**Affiliations:** Department of Biophysics and Radiobiology, Biological Sciences Centre, Federal University of Pernambuco, Recife, PE, Brazil; Department of Immunology, Aggeu Magalhães Research Institute (IAM), Oswaldo Cruz Foundation (FIOCRUZ), Recife, PE, Brazil

**Figure:**
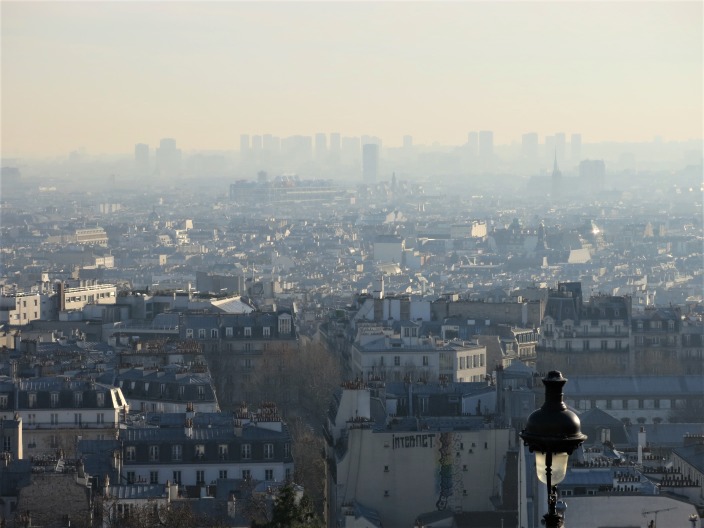
Photo: Pollution event in Paris, 8 December 2016 – Paris seen from the hill of Montmartre (Source: Wikimedia Commons, released into the public domain worldwide).

Despite some advances, poor air quality in Europe persists, even in high-income countries. According to the latest report from the European Environmental Agency (EEA), more than 500 000 people died in Europe in 2015 due to air pollution [1]. This corresponds to about one sixth of all deaths related to air pollution in the world. The EEA data take into account deaths caused by PM_2.5_ (particulate material with less than 2.5 μm in diameter), NO_2_ and ozone. Particulate material has been recognized as the main risk factor associated to air pollution. About 83% of all deaths related to air pollution in Europe in 2015 were attributed to PM_2,5_, 14% to NO_2_ and the remaining deaths were attributed to ozone. An analysis of the countries with more deaths attributed to air pollution reveals that Italy, Germany, Poland, France and UK, in this order, are at the top of the list (**Table 1**). Spain, Romania, Bulgaria, Greece and Hungary complete the 10 countries with more deaths caused by air pollution. If we look at the countries with higher death rates, the list changes considerably, including Eastern and Southern European countries as the leading ones, with Kosovo, Bulgaria, Serbia, Macedonia and Hungary at the top of the list (**Table 2**) and also includes Italy, Greece, Romania, Poland and Croatia. These data show that despite stricter rules for car emissions, there are unjustifiable high numbers of deaths related to air pollution in many European countries. The annual mean PM_2.5_ levels for most of the countries with higher death rates are close to or twice as high as the WHO recommended levels of 10 μg/cm^3^, and all the countries listed in **Table 1** and **Table 2**, except UK, show PM_2.5_ levels above this limit.

**Table 1:** European countries with more deaths attributable to air pollution, according to the Air Quality Report 2018

Rank	Country	Premature deaths [1]	Annual mean PM_2.5_* (μg/m^3^) [1]	% Diesel vehicles in use [2]	Average age of vehicles [3]	% Energy produced from coal [4]
1	Italy	84 300	18.5	41.1	10.7	16.7
2	Germany	78 400	12.3	32.2	8.9	45.8
3	Poland	47 500	21.6	29.4	17.2	80.9
4	France	47 300	11.9	69.8	9.0	2.2
5	United Kingdom	41 490	9.4	37.2	8.5	22.9
6	Spain	38 600	12.7	56.6	11.4	16.5
7	Romania	27 280	18.1	37.0	15.3	27.6
8	Bulgaria	15 190	24.1	48.4	NA†	46.2
9	Greece	14 910	19.1	4.9	13.5	42.7
10	Hungary	14 630	18.9	27.8	14.5	19.5
11	Serbia	14 280	23.3	NA	NA	72.4
12	Netherlands	11 990	12.3	16.5	9.5	37.3
13	Czech Republic	11 050	17	35.3	14.5	54.0
14	Belgium	9120	13	61.5	8.8	6.3
15	Austria	7480	13.3	56.9	8.9	8.2

*PM_2.5_ – particulate material with less than 2.5 μm in diameter.

†NA – not available.

**Table 2:** European countries with higher death rates attributable to air pollution, according to the Air Quality Report 2018

Rank	Country	Deaths by 100 000 inhabitants [1]	Annual mean PM_2.5_ (μg/m^3^)* [1]	% Diesel vehicles in use [2]	Average age of vehicles [3]	% Energy produced from coal [4]
1	Kosovo	215.5	26.4	NA†	NA	97.5
2	Bulgaria	210.9	24.1	48.4	NA	46.2
3	Serbia	200.7	23.3	NA	NA	72.4
4	Macedonia	154.7	28.7	42.5	NA	58.4
5	Hungary	148.4	18.9	27.8	14.5	19.5
6	Italy	138.7	18.5	41.1	10.7	16.7
7	Greece	137.3	19.1	4.9	13.5	42.7
8	Romania	137.3	18.1	37.0	15.3	27.6
9	Poland	125.0	21.6	29.4	17.2	80.9
10	Croatia	122.1	17.4	43.3	14.1	20.6
11	Montenegro	110.9	18.5	NA	NA	50.3
12	Bosnia and Herzegovina	105.1	18.9	52.2	NA	64.0
13	Czech Republic	104.9	17	35.3	14.5	54.0
14	Slovenia	99.9	17.4	43.9	11.2	29.6
15	Slovakia	99.8	19.1	7.9	13.4	11.9

*PM_2.5_ – particulate material with less than 2.5 μm in diameter.

†NA – not available.

Especially in the case of Italy, Germany, France and UK, the high number of deaths related to air pollution might be related to the high number of diesel cars [2], which account for 30 to almost 70% of all vehicles registered in these countries (**Table 1**). This is also true for many Eastern and Southern European countries such as Bulgaria, Macedonia, Lithuania, Croatia and Latvia. Another factor that contributes to air pollution-related deaths is the use of coal for electricity generation. Among the fuels used in power stations, coal is the most polluting one, releasing more particulate material than any other fossil fuel. The percentage of electricity generated by coal burning is low in France (Table 01), which relies mostly on nuclear power. However, its importance in UK, Germany, Greece, Czech Republic and particularly Poland, Serbia and Macedonia is much higher, reaching at least 60% in the later countries [4]. In Kosovo, almost all the electricity is produced from coal.

Air quality is also highly affected by the age of the vehicle fleet, since older cars have higher emission levels. On the list of countries with higher death rates, the average age of vehicles is above 13 years in most of the countries [3], with predominance of more polluting Euro 3 and Euro 4 vehicles. This is especially the case of Poland, Romania, Hungary and Czech Republic with an average age of vehicles over 14 years. Countries with the lowest death rates show an average vehicle age below 9 years (data not shown), with predominance of vehicles that have, at least, Euro 5 emission standards. Older cars reflect directly on air quality: all the countries in which they are more abundant have also the highest PM_2.5_ annual means. During many years, the import of used cars, especially to Eastern and Southern European countries, has been very common and certainly contributed to the poorer air quality in these countries [5]. Some countries are taking measures to restrict the age of used imported cars [6]. However, the Volkswagen emission scandal may have even worsened this problem, since countries with stricter environmental rules allowed sending cars involved in the scandal to other countries [7].

Another factor that contributes to air pollution in Europe, but only recently has received more attention, is related to shipping emissions. The contribution of shipping emissions to particulate material air pollution varies according to the fuel type, diesel or heavy fuel oil. Diesel used in ships is already more pollutant than the one used in heavy vehicles due to less stringent regulations. Heavy fuel oil is even more pollutant due to a much higher sulphur content than ordinary diesel. Shipping emissions are also affected by the region analysed and the method used on the study [8] and is more important in countries closer to the busy shipping routes of North Sea and the Mediterranean region. In the North Sea, the contribution of shipping emissions to PM_2,5_ levels may be as high as 5% in the Netherlands and 4% in UK. In the Mediterranean region, shipping emissions have been found to increase PM_2,5_ levels by as much as 14% in the Gibraltar Strait and 6% in Barcelona (8). PM_1_ (particulate material with less than 1.0 μm in diameter) levels were increased by 20% in Genoa during summer that, according Viana et al. [8], is probably due to the increase in the number of passenger ships in this period.

Reducing the levels of air pollution is complex and involves public awareness, policy measures, and technological advances. In order to reduce air pollution levels, some Eastern European countries have proposed restrictions or even the ban of diesel cars, especially after the Volkswagen emission scandal [9], along with the implementation of low and ultra-low emission zones at city level. This energy transition is highly dependent on zero-emission cars development and adoption, what has been happening in a much higher speed in Norway, for example, where electric cars currently represent about 60% of all new car sales [10]. In Norway, as well as in most of the other countries where electric cars are significantly rising in sales, public incentives through lower taxes, besides other measures, have been a key factor for this change, which has been accompanied by an increase in public awareness, further catalysed by the Volkswagen emission scandal. The European Air Quality Index, launched last year, is also an important tool to increase awareness about air pollution among the European population [11]. However, much more needs to be done in order to reduce the number of deaths caused by air pollution in Europe. Regarding shipping emissions, the strictest fuel regulations, limiting sulphur content of shipping fuels, besides other parameters, are supposed to be adopted in 2020. These regulations are fundamental to reduce particulate material as well as SO_x_ and NO_x_ derived from shipping emissions [12]. While the increase in the number of electric cars is also fundamental for reducing air pollution levels in Europe, this needs to be accompanied by a complete energy matrix transition, which involves banning coal use. The use of coal for electricity generation has been falling in UK and Germany, for instance, and both countries have plans to phase out coal: UK in 2025 [13] and Germany between 2035 and 2038 [14]. Nevertheless, while the decrease in coal use has been faster in UK, Germany, the first economy in Europe and forth in the world, is still highly dependent on coal. Additionally, the high coal dependence in many Western and Southern European countries, which have been using cheap coal energy to speed up economic development, is a major issue that also needs to be addressed. It is time to speed up coal ban in favour of cleaner energy sources, a measure that, along with restrictions in the use of fossil fuels in vehicles, will help to improve air quality and reduce deaths caused by air pollution in Europe.
